# Rehydration during Endurance Exercise: Challenges, Research, Options, Methods

**DOI:** 10.3390/nu13030887

**Published:** 2021-03-09

**Authors:** Lawrence E. Armstrong

**Affiliations:** Human Performance Laboratory and Korey Stringer Institute, University of Connecticut, Storrs, CT 06269-1110, USA; Lawrence.Armstrong@Uconn.edu

**Keywords:** thirst, drinking, sweat, sodium, hyponatremia, overhydration, dehydration, marathon, triathlon

## Abstract

During endurance exercise, two problems arise from disturbed fluid–electrolyte balance: dehydration and overhydration. The former involves water and sodium losses in sweat and urine that are incompletely replaced, whereas the latter involves excessive consumption and retention of dilute fluids. When experienced at low levels, both dehydration and overhydration have minor or no performance effects and symptoms of illness, but when experienced at moderate-to-severe levels they degrade exercise performance and/or may lead to hydration-related illnesses including hyponatremia (low serum sodium concentration). Therefore, the present review article presents (a) relevant research observations and consensus statements of professional organizations, (b) 5 rehydration methods in which pre-race planning ranges from no advanced action to determination of sweat rate during a field simulation, and (c) 9 rehydration recommendations that are relevant to endurance activities. With this information, each athlete can select the rehydration method that best allows her/him to achieve a hydration middle ground between dehydration and overhydration, to optimize physical performance, and reduce the risk of illness.

## 1. Introduction

The essential components of central nervous system maintenance of body water volume and concentration include perceptions, behavior, nervous system responses, and the release of hormones (vasopressin, AVP; angiotensin II; atrial natriuretic peptide; apelin) [1,2,3,4]. Perturbations of whole-body water volume and concentration are monitored by the brain, the resulting thirst and oropharyngeal sensations modulate drinking, and neuroendocrine responses regulate water and electrolyte excretion or retention by the kidneys [1]. During typical daily activities that do not include exercise, these complex interactions act to maintain total body water volume and serum concentration within 1–3% of baseline each day [5,6,7]. However, the relative influence of these processes varies with different life activities [8]. Table 1 explains that, during sedentary daily pursuits in a mild environment, renal responses and thirst are the primary homeostatic regulators. During prolonged endurance exercise at low intensities (5–24 h duration), renal responses and thirst have minor-to-large effects on water regulation. As the duration of exercise increases, sweat losses become a major factor in whole-body water balance [9], regardless of the volume of fluid consumed.

**Figure 1:**
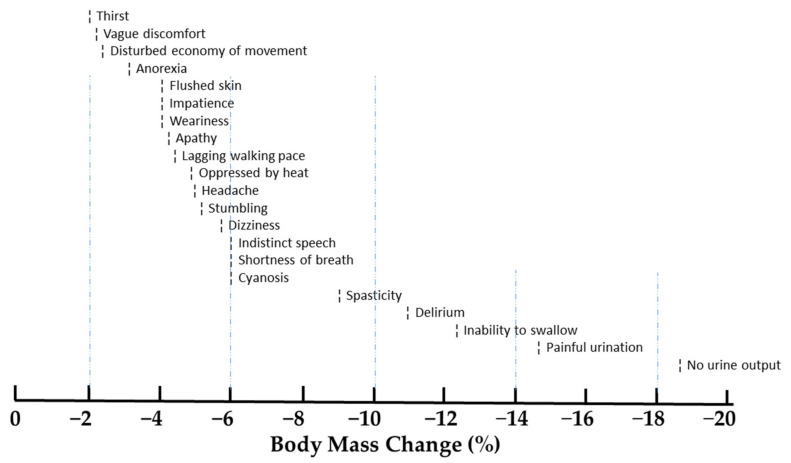
Signs and symptoms of dehydration in men who walked in the desert without drinking. The symbol which appears to the left of each sign or symptom identifies the approximate water deficit of its first report. Based on information from [12].

This review article considers endurance exercise from the perspectives of body water and electrolyte balance, the negative effects that substantial fluid–electrolyte disturbances (i.e., both water loss and gain) have on competitive performance and health, and ways that endurance athletes can minimize performance decrements and mitigate the risk of exercise-associated illness. This is important because an endurance athlete can lose as much as 11–12% (7.8–8.5 kg) of body weight in the form of water, during a 12.3-h Ironman triathlon in a cool environment (3.8-km swim, 180-km bike, 42.2-km run) [10]. This also is important because day-long walking or hiking in a desert environment can result in extreme body mass losses of 14–18% when fluids are unavailable or restricted (Figure 1). Conversely, excessive fluid intake (i.e., water retention) can result in a body mass gain of more than 10% (7.8 kg) in 12.7 h while competing in an ultraendurance triathlon (ambient temperature, Tamb, 20.5 °C) [11]. These vastly different changes of body mass represent the primary problem and focus of this review paper: how to maintain a rehydration middle ground during prolonged exercise that reliably reduces the risk of illness by avoiding overhydration, and maintains exercise performance by avoiding significant dehydration.

## 2. Problem: Water and Salt Losses during Endurance Exercise

**Figure 2:**
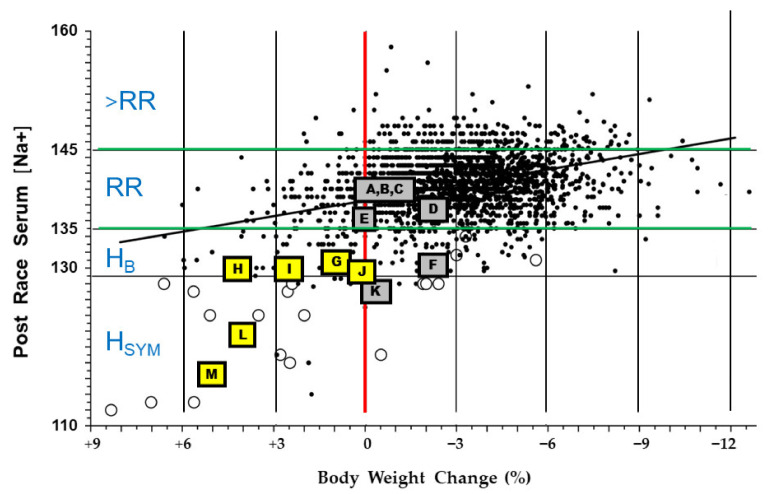
The relationship between body weight change (%) and serum Na^+^ after 4.0–13.3 h of exercise (*n* = 2135) as modified from [20]. Solid circles (●) represent asymptomatic marathon runners and Ironman triathletes. Open circles (○) depict athletes with severe symptoms including hyponatremic encephalopathy (central nervous system dysfunction due to brain swelling). Horizontal zone abbreviations: >RR, serum Na^+^ concentration above resting normal; RR, the laboratory reference range for healthy adults (green horizontal boundaries); H_B_, biochemical hyponatremia which involves few or no symptoms; H_SYM_, symptomatic hyponatremia. Symbols A–M were overlaid by the present author (see details below in Section 3.2.1). Gray highlighted symbols depict individuals with fluid intake rates of ≤700 mL/h and body mass losses of 0.1 to 2.6%. Yellow highlighted squares indicate exertional hyponatremia cases (each *n* = 1) with fluid intake rates ranging from 733–2061 L/h and body mass increases of +0.1 to +5.0%. Reprinted via the PNAS Open Access option from [20].

Most ultraendurance competitors do not meet their fluid needs during competition [13], due primarily to three factors that interact to influence sweat volume and body mass during prolonged exercise [14]. The first of these factors is exercise intensity. Table 2 presents whole-body water balance measurements of 32 cyclists who completed a 164-km event in the state of Texas, USA during the month of August [15]. Cyclists have been grouped on the basis of time to complete 164 km: 9.6, 6.3, and 4.8 h. The total volume of sweat lost by these groups were similar (range of 7000–7200 g), demonstrating that the higher exercise intensities of groups 4.8 and 6.3 stimulated a greater sweat rate per hour (*p* < 0.01 to 0.0001) than that of group 9.6. Exercise intensity also affected body mass proportionally. The body mass change values for cyclist groups 9.6, 6.3, and 4.8 (columns 2–4, row 11) were −1800, −2300, and −2750 g. The second factor is exercise duration. As shown in Table 2, slower cyclists may be on the course at least twice as long as faster competitors. Not surprisingly, similar body mass losses occur commonly (Figure 2) during ultra-running, ultra-cycling, and ultra-triathlon events [16]. Environmental temperature represents a third factor that influences body water balance. During 42.2 km marathon running, mild ambient conditions of 7, 10–12, and 20 °C resulted in mean sweat rates of 0.81, 0.96, and 1.52 L/h, respectively [17]. In addition, researchers measured the sweat rates of athletes in a laboratory building (29 °C, 51% relative humidity; running and cycling protocols), a mobile laboratory (29 °C, 65% rh), or field environment (25 °C, 55% rh) [18]. The majority of these athletes competed in team/skill sports (*n* = 1022) and individual endurance sports (*n* = 255). The highest average sweat rates were observed in the sports of American football (1.51 L/h) and Endurance Sports (1.28 L/h), whereas the lowest occurred in baseball (0.83 L/h) and soccer (0.94 L/h).

Representing extreme points of reference, the following individual sweat rate values have been observed. First, an elite marathon runner (age, 26 y; height, 185 cm; body mass, 66.9 kg) produced 3.7 L of sweat per h during the 1984 Los Angeles Summer Olympics marathon (24–28 °C Tamb; time to complete 42.2-km race, 2 h 14.3 min; body mass change, 5.43 kg, −8.1%) [21]. Second, a runner (age, 30 y; height, 185 cm; body mass, 91.8 kg) who had experienced heatstroke twice previously was observed to have a sweat loss of 4.1 L during a 70-min laboratory race simulation (25 °C) [22]. Third, the sweat rate of a male tennis player (age, 26 y; height, 197 cm; body mass, 91.4 kg) was 4.3 L/h during a 60 min laboratory simulation (36.1 °C; brisk walking and jogging on a treadmill) [23]. The sweat sodium losses (i.e., calculated by multiplying the measured sweat volume times the sweat sodium concentration) during the two simulations were 5930 and 7610 mg Na+, respectively. Additionally, as noted above, extreme body mass losses of −11 to −12% were experienced by competitors in an Ironman triathlon that required an average of 12.3 h to complete [10].

### 2.1. Effects of Dehydration on Endurance Exercise Performance

**Figure 3:**
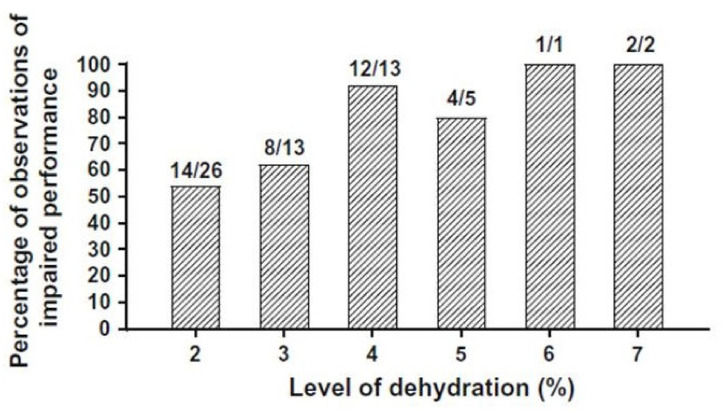
The effects of dehydration on exercise performance. Fractions represent the number of statistically significant (*p* < *0*.05) observations out of the number at each level of body mass loss. Across all dehydration levels, 68% of comparisons indicated impairment. Reprinted from [32] via the Creative Commons Attribution 4.0 International License (http://creativecommons.org/licenses/by/4.0/) accessed on 2 March 2021.

The negative influences of dehydration on exercise performance are recognized by professional sports medicine organizations [24,25,26,27] and sport governing bodies [28] in position statements regarding rehydration, exertional heat illness, and physical performance. Although the precise water deficit at which performance decrements occurs is difficult to determine because of inter-individual differences, there is overall consensus in the literature that dehydration of −2 to −4% represents the range in which endurance exercise performance declines [24,25,26,28,29,30,31]. This effect is illustrated in Figure 3, which presents a performance analysis of 34 published studies involving dehydration; a body water deficit of 1–3% was less likely to impair endurance exercise performance significantly (*p* < 0.05) than dehydration of 4–7% [32]. These 60 statistical comparisons involved outdoor track running, trail running, outdoor road cycling, indoor treadmill running, indoor rowing ergometry, and indoor cycle ergometry.

It also is relevant that controlled field studies have reported statistically significant decrements of endurance exercise performance at lower levels of dehydration (−1.0 to −2.2%). These controlled protocols evaluated running competitions at (a) three distances on an outdoor all-weather track [33] and (b) an outdoor cycling hill-climbing performance competition [34]. Similar performance decrements were reported for two indoor cycling simulations [35,36] when dehydration ranged from 1.0–1.8%.

Decrements of outdoor cycling performance which occur at ≤4% body mass loss have been disputed in one meta-analysis [37] which evaluated 5 research articles (13 effect estimates). However, virtually all researchers agree that moderate-to-severe dehydration (e.g., 4–7% versus 1–2% body mass loss; Figure 3) results in greater physiologic strain and decreased aerobic exercise performance [24,26,29,30,32,38,39,40,41].

Physiologists agree that dehydration-induced aerobic performance decrements are greater in hot versus cool environments [24,29,41,42], due to greater thermal and cardiovascular strain (i.e., increased skin temperature and blood flow, greater plasma volume loss, decreased cardiac output) that becomes physiologically impactful in the dehydration range of 2–4% of body mass [43,44]. In terms of associated physiological effects, a summary of 8 relevant publications [29] noted that a 4–5% dehydration level reduced maximal oxygen consumption (VO_2max_) more in a hot environment (−9 to −27% when Tamb ≥ 30 °C) than in a cool or mild environment (−3 to −7% when Tamb was 15–26 °C). The consequences of this dehydration included a shorter exercise time to exhaustion, an obligatory reduction in exercise intensity, or both.

### 2.2. Effects of Dehydration on Symptomatology and Illness

The classic desert field observations of Adolph and colleagues [12] during the mid-1940s were among the first descriptions of exercise-induced dehydration that approached and exceeded 10% body mass loss. Although Adolph’s systematic observations of dehydrated soldiers walking in the desert (Tamb > 37 °C) were not subject to statistical analyses, they provide a thorough description of the detrimental health effects of prolonged dehydration. Figure 1 presents a summary of the signs and symptoms that were reported when dehydration exceeded 2%. The following Section 2.2.1 through Section 2.2.3 describe disorders that result from water and salt deficiencies.

#### 2.2.1. Exercise Associated Collapse

A patient classification and care matrix, developed after years of treating runners in the medical tent of the Twin Cities Marathon, Minnesota, USA [45] facilitates clinical decision making and expedites the transition of distressed runners through a field medical facility near the finish line. Treatment of exercise-associated collapse centers on fluid replacement and body cooling or warming if needed. For suspected dehydration after the marathon, oral fluid replacement is preferred (e.g., water, fluid-electrolyte beverage, or 200–300 mL of salty soup bouillon to aid fluid retention). Intravenous fluid is necessary when casualties are unable to tolerate oral intake, and when there are clinical indications of severe volume depletion or ongoing fluid losses from vomiting or diarrhea. During medical treatment, to avoid adding water to a runner who already is overhydrated, exertional hyponatremia (see Section 3.2) is ruled out before intravenous fluid administration by assessing the serum Na^+^ concentration of runners who finish after 4 h [45].

#### 2.2.2. Exertional Heat Illnesses

Water and salt losses in sweat have implications for the health of athletes who exercise in hot environments. Water and sodium deficits are recognized as predisposing factors for exertional heat stroke [46,47,48], exertional heat cramps [49], and exertional heat exhaustion [43,50]. Regarding the latter illness, research has shown that (a) mild exercise (40–50%VO_2max_) in hot environments (34–39 °C) does not induce heat exhaustion unless a significant fluid-electrolyte loss and cardiovascular strain exist; (b) a moderate but cumulative dehydration across 3 days can result in exertional heat exhaustion [51,52]; and (c) 85% of heat exhaustion patients who presented at a deep metalliferous mine infirmary exhibited a urine specific gravity of 1.020–1.040, indicating mild-to-severe dehydration [53].

#### 2.2.3. Kidney Dysfunction and Renal Stress

Failure of the kidneys to perform essential functions (i.e., the clinical disorder named acute renal failure) is possible but uncommon during marathon footraces of 42.2 km and dehydration less than 4% of body mass [54]. However, both immediate (during and post-race) and delayed effects (i.e., 1–5 d after the event) of dehydration have been reported as abnormal values for urine flow rate, osmolar clearance, creatinine clearance, and protein in the renal filtration apparatus [54]. When no renal dysfunction is observed, considerable renal concentrating stress is possible. For example, 11 out of 33 cyclists who finished a summer 164 km ultraendurance event exhibited marked urine concentration, verified with specific gravity > 1.030 [55].

## 3. Problem: Overhydration during Endurance Exercise

### 3.1. Hyperhydration and Exercise Performance

No evidence suggests that deliberate pre-exercise consumption of excess pure water has an ergogenic effect on exercise performance [24,56]. Glycerol, however, often is ingested prior to exercise (e.g., 1.2 g/kg body mass with a volume of fluid equal to 26 mL/kg body mass) to hyperhydrate athletes by increasing water retention and plasma volume while decreasing urine volume [57]. This act delays reaching a level of dehydration that degrades exercise performance. Research results are not conclusive, in that studies have shown both an ergogenic effect and no effect on exercise performance [58,59]. However, one caveat should be noted. As glycerol dilutes both intracellular and extracellular fluids prior to exercise, it may predispose athletes to low serum Na^+^ as described in the next paragraph, especially if aggressive drinking occurs during exercise. A similar predisposition to low serum Na^+^ has been reported for deliberate overhydration with water and dilute fluids [24,60,61].

### 3.2. Exertional Hyponatremia (EHN): A Potential Medical Emergency

When overhydration during exercise dilutes blood, an osmotic pressure gradient causes water to move into cells. The resulting cell swelling can result in EHN, one of the few illnesses that is potentially fatal to otherwise healthy athletes during exercise. Recognizable symptoms appear in most athletes at a serum concentration of approximately 130–135 mmol Na^+^/L [26,62] and include lightheadedness, dizziness, nausea, puffiness (e.g., hands and feet), and body weight gain from baseline [63]. The majority of athletes whose serum Na^+^ is below 130 mmol/L (Figure 2) experience symptoms; these may include headache, vomiting, frothy sputum, difficulty breathing, pulmonary edema (i.e., fluid accumulation with swelling), and altered mental status such as confusion or seizure that results from cerebral edema [10,64].

The signs and symptoms of EHN do not necessarily correlate with the serum Na+ in the range shown in Table 3 (≥130 mmol Na^+^/L). The total symptoms score (column 10), rated with a validated Environmental Symptoms Questionnaire [65], was not related to the change of serum Na^+^ (column 5). Indeed, the self-rated symptoms of hyponatremic cyclists LC and AM (serum Na+ of 130 mmol/L) ranked among the lowest in this subject sample [66]. Thus, the severity of symptoms and not the absolute value of serum Na^+^ concentration guide the course of medical treatment [63].

Table 3 includes data regarding two recreational cyclists (LC and AM) who began a summer 164-km event (Tamb, 34 ± 5 °C) with normal serum electrolytes but finished the ride with a serum sodium concentration of 130 mmol/L, indicative of mild EHN. The data of 12 other finishers (A–L, column 1) are presented to allow comparisons. Although they did not ride together, both cyclists consumed a large and similar relative volume of fluid (191 and 189 mL/kg), experienced an identical 11 mmol/L decrease of serum sodium, and reported low thirst sensations. However, one (LC) gained 3.1 kg (+4.3% of body mass) during 8.9 h of exercise (i.e., suggesting a dilutional effect) and the other (AM) maintained body mass (+0.1 kg, +0.1%, 10.6 h), suggesting that no excess fluid was retained. Thus, Table 3 suggests a complex, individualized EHN etiology [66].

After exercise, fluids should be consumed judiciously because symptoms of EHN may develop hours after excess fluid consumption, as described in two published case reports. The first involved a 21-year old man who had aggressively consumed water and gained 5.25 kg of body weight during 5 h of treadmill exercise in a hot environment [61]. He was asymptomatic until he experienced nausea and malaise late that evening, and was transferred to a nearby hospital with a serum Na^+^ of 122 mmol/L. After a night of observation, fluid restriction, and large urine output, he was released at 11:00 a.m. the next morning without symptoms. A more serious EHN case with delayed symptom onset involved a 49-year old runner who finished a 42.2 km marathon in 4 h 22 min then boarded an airline flight to return home [67]. Approximately 5 h after he finished the race, he became ill and experienced a grand mal seizure in the aisle of the cabin. The pilots diverted the aircraft to a nearby city for an emergency landing. The runner was transported to a hospital, where he experienced two additional seizures while unconscious with a serum Na^+^ of 129 mmol/L. A chest x-ray indicated pulmonary edema and a brain scan revealed cerebral edema; he also was diagnosed with renal insufficiency and liver damage. During the ensuing 18 months, this runner learned that he was unable to mentally process information that had previously been routine, and he was unable to perform his professional duties.

#### 3.2.1. Predisposing Factors for EHN

Exercise duration greater than 4 h, high sweat rate, high sweat Na^+^, and small body size have been identified as predisposing factors for EHN [63,68,69,70]. Table 4 allows consideration of other risk factors; data are rank-ordered on the basis of final serum Na^+^ (column 5). As the severity of EHN increased (i.e., moving from top to bottom as serum Na^+^ decreased), both body mass change (column 6) and the rate of fluid intake (column 8) trended toward increasing. The individuals and groups in Table 4 (labeled A through M in column 1) also are depicted in Figure 2, allowing comparisons to a large data base of marathon runners and Ironman triathletes. The open symbols (○) in Figure 2 represent individuals who sought medical care for symptomatic EHN.

A few athletes possess a “perfect storm” of characteristics in which a high sweat rate (e.g., 2.0–3.0 L/h) coexists with a high sweat sodium concentration (e.g., 40–80 mmol Na^+^/L; 2.3–4.6 g NaCl/L). These athletes may be identified by white salt deposits on a shirt, jersey, or shorts. Due to their relatively large sodium loss in sweat, they are at increased risk of developing EHN. In a hypothetical calculation, Hiller [68] noted that an Ironman triathlete with a sweat rate of 1.5 L/h could lose 36 g of NaCl (14,040 mg of sodium) in 12 h. This observation is supported by the mathematical prediction model of Montain et al. [76], which demonstrates that a high sweat sodium concentration is an important etiological factor.

#### 3.2.2. EHN Etiologies

Multiple EHN origins have been described [20,61,66,67,71], and consensus regarding a single etiology is difficult to reach because some cases reportedly involve hyponatremia with body mass gain (i.e., hypervolemic hyponatremia, water retention that exceeds sweat and urine losses) [10,77,78,79], whereas other EHN cases involve hyponatremia with body mass loss due to partially replaced sweat water and sodium losses (hypovolemic hyponatremia) [10,68,77,80]. Importantly, a 2015 consensus document noted that all known EHN fatalities to that date had involved overconsumption of dilute fluids [63].

#### 3.2.3. EHN Cases Involve Variable Vasopressin Responses

Vasopressin (antidiuretic hormone) is the body’s principal water-regulating hormone. It functions to maintain body water balance, in conjunction with thirst, by regulating serum osmolality within narrow limits [81]. Although dehydration with elevated serum osmolality is the primary stimulus for the release of vasopressin from the pituitary, non-osmotic factors also are known, including plasma volume decrease, hypoglycemia, nausea, and vomiting [82].

In an unknown percentage of EHN cases, serum vasopressin increases abnormally during overhydration, facilitating water retention. This inappropriately high serum vasopressin also stimulates sodium excretion by the kidneys, reducing serum Na^+^. As vasopressin has a brief half-life and laboratory analysis is technically difficult, this hormone rarely is analyzed in cases of EHN. Nevertheless, this condition was verified during a marathon field study [83] in which 43% of runners with a serum Na^+^ < 130 mmol/L exhibited inappropriately high serum vasopressin levels (range, 3–17 pg/mL), and during a case of symptomatic EHN that developed during a laboratory investigation [61]. A notable exception to these reports occurred among 7 symptomatic Ironman triathletes [74] who finished 12 h of exercise with a median body weight loss of 0.5%, post-exercise plasma Na^+^ of 128 mmol/L, and a post-exercise plasma vasopressin concentration of 1.6 pmol/L; these values represented symptomatic EHN with a low serum vasopressin concentration. A control group of 11 asymptomatic triathletes exhibited the following comparison values: body weight loss of 3.9%, plasma Na^+^ of 141 mmol/L, and a plasma vasopressin concentration of 4.6 pmol/L; these levels represented a typical hormonal response to moderate dehydration. These variable vasopressin responses illustrate why it is difficult to attain consensus regarding the role of arginine vasopressin in EHN.

A coherent explanation for variable vasopressin responses was published by Hew-Butler [82]. Her review article distinguished intense (brief, >90% of maximal oxygen consumption), steady state (sustained at 40–60% of maximal oxygen uptake), and prolonged endurance (>1 h) exercise. During the former, an obvious and statistically significant increase of plasma vasopressin occurs that exceeds the expected increase due to increased plasma osmolality. Steady state exercise also generally stimulates a statistically significant vasopressin increase. During prolonged competitive endurance exercise, a similar vasopressin increase occurs, with or without significant increases of serum Na^+^ or osmolality; this elevation persisted for 2 h after a 24 h competitive track race and for 31 h after a 38 km non-competitive run. In all of these exercise types, it appears that published vasopressin responses are difficult to interpret because of differences in exercise intensity [82].

#### 3.2.4. Evidence for an EHN Drinking Rate Threshold

A previous analysis of 6 groups of runners and triathletes reported that no case of symptomatic EHN occurred (serum sodium < 130 mmol/L during continuous exercise that lasted 3.2–12.3 h) among 270 athletes who consumed less than 750 mL of fluid per hour [67]. In addition, case reports of two female ultradistance triathletes [73] observed that their post-race hyponatremia (130 and 131 mmol Na+/L) was accompanied by weight gain (0.5 and 1.5 kg). They consumed fluids during competition at a rate of 730 and 760 mL/h. Figure 2 also supports this drinking rate threshold for the onset of EHN, in that all yellow highlighted square symbols represent individuals who consumed fluids at a rate > 700 mL/h, and gained weight during their respective exercise bouts. These individuals are clustered in the lower left quadrant of Figure 2, among symptomatic athletes (open symbols, ○). These observations suggest that endurance athletes who consume fluids at a rate < 700 mL/h have a decreased risk of EHN. This theoretical 700 mL/h fluid consumption rate threshold is consistent with the 400 to 800 mL/h recommendations of both the International Marathon Medical Directors Association [84], the American College of Sports Medicine [24], and a mathematical model that was designed to clarify the etiology of EHN [76].

#### 3.2.5. Does Sodium Intake Counteract a Low Serum Na^+^?

It is widely recognized that salt (sodium chloride, NaCl) capsules are consumed during triathlons and marathons. Exploring this trend within endurance sports, Hoffman & Stuempfle [80] reported that 90–96% of runners consumed sodium supplements during a 161-km ultramarathon footrace because they believed that it prevented muscle cramps and hyponatremia. However, there is little evidence to support this belief. For example, Table 3 shows that cyclists J, K, L, LC and AM consumed the largest amounts of sodium but experienced the greatest decrease of serum Na^+^, whereas cyclists A, B, C and D consumed small amounts of sodium and experienced an increased serum Na^+^ [61]. Therefore, sodium consumption did not prevent EHN from occurring in cyclists LC and AM, and low sodium intake by other cyclists was not associated with EHN. Similar conclusions have been published regarding ultramarathon competitors by Speedy et al. [85], Hew-Butler et al. [86], Hoffman and Stuempfle [80], and Hoffman and Myers [87].

Two controlled laboratory studies also have quantified the effects of sodium consumption on serum Na^+^. The first provided 3911 mg of sodium during 6 h of exercise in a 34 °C environment [88], and the second provided 1409 mg sodium during 3 h of exercise at 30 °C [89]. Post-exercise measurements detected a mean serum Na^+^ increase of 3 mmol/L (i.e., supplemented versus control experiments) in both studies, indicating that sodium supplementation had a minor influence on serum Na^+^ levels.

## 4. The Complexity of Thirst and Drinking

Thirst prompts seeking and consuming water, and is measured with a subjective rating scale. Drinking behavior is distinct from thirst, is measured as the volume of fluid consumed, and may involve fluid selection on the basis of preferred or required characteristics (i.e., temperature, palatability, ingredients, energy content) [90].

During sedentary daily activities (i.e., producing a small 24-h water turnover), the perception of thirst, the act of drinking, renal regulation of water and electrolytes, and neuroendocrine responses (Table 1) adequately regulate total body water volume and serum concentration within 1–3% of each individual’s baseline, from one day to the next [5,6,7]. During prolonged endurance exercise, however, the relationship between perception of thirst and whole-body fluid–electrolyte balance can be distorted by physiological challenges [32,91] such as sizeable water and sodium losses in sweat, movement of water between the intracellular and extracellular spaces, or plasma volume depletion [6].

### 4.1. Multiple Factors Influence Drinking during Endurance Exercise

**Figure 4:**
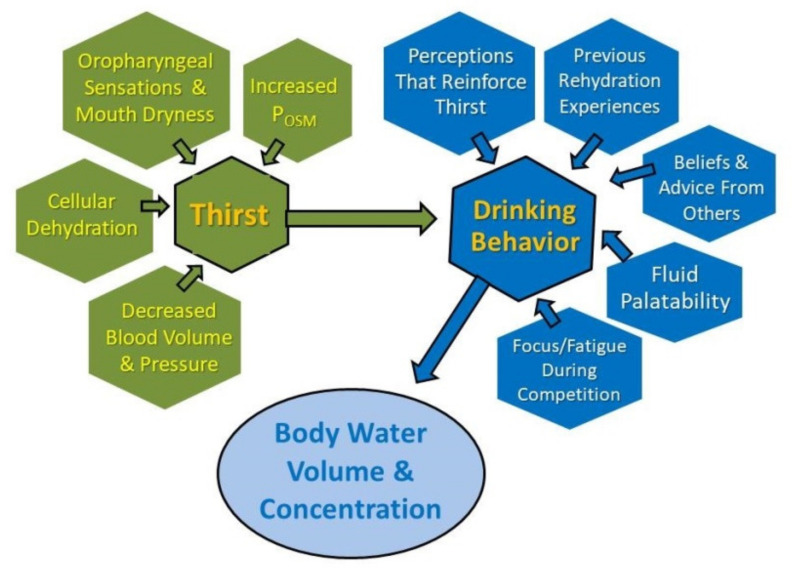
Influences on thirst and drinking behavior during endurance exercise. All factors in this diagram are perceived, monitored, and/or regulated by the brain.

Figure 4 presents several factors that influence thirst and drinking behavior, each of which is monitored and regulated continuously by the central nervous system. Thus, the phrase dynamic complexity applies to the vast, integrated, brain-wide network of nerve circuits and brain regions that regulate thirst, drinking, body water volume, and fluid concentration [92]. The findings of two recent human studies [93,94] suggest that thirst is one of multiple conscious perceptions and subconscious autonomic responses (Figure 4) that evolve simultaneously during dehydration and rehydration to influence drinking behavior. Simply stated, the internal motivation to consume water is influenced by multiple factors that reinforce the perception of thirst.

**Figure 5:**
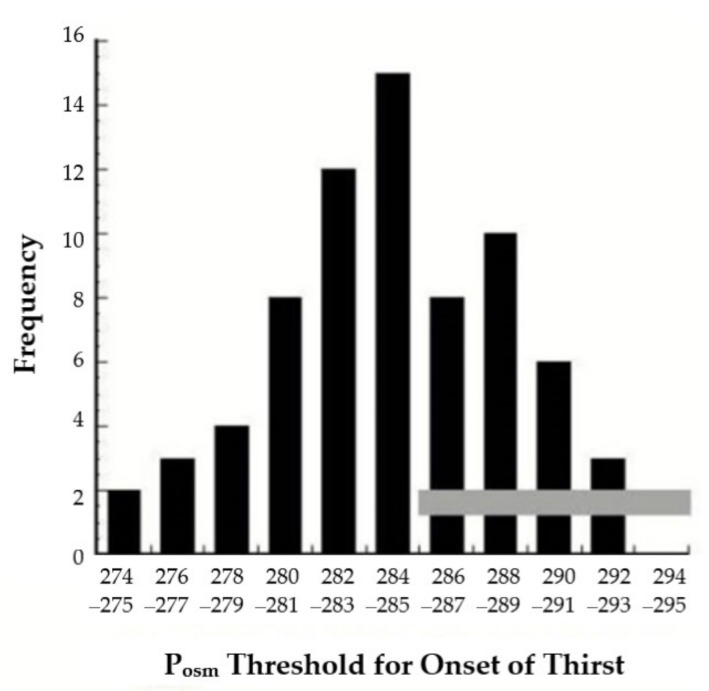
Frequency distribution of the plasma osmolality (P_osm_) threshold for the onset of thirst. The horizontal gray bar delineates the laboratory reference range of P_osm_ values (285–295 mOsm/kg) for healthy adults. Reprinted under the terms and conditions of the Creative Commons Attribution (CC BY) license (http://creativecommons.org/licenses/by/4.0/) accessed on 2 March 2021. Modified from [97].

As dehydration concentrates extracellular fluid, plasma osmolality (P_osm_) is recognized as the primary factor that stimulates thirst [2,81,95]. However, as shown in Figure 5, the P_osm_ threshold at which thirst is perceived varies greatly across individuals (range, 274–293 mOsm/kg) and may be lower than the range of laboratory reference values for P_osm_ (285–295 mOsm/kg) [96]. The data in Figure 5 were compiled from 5 published human studies; the shape of this frequency distribution implies that the thirst threshold is a multifactorial (polygenic) characteristic [97]. Although inter-individual differences of the P_osm_ threshold for thirst have not been studied during exercise, Figure 5 suggests that the level of dehydration (i.e., increased P_osm_) that initiates drinking during exercise might differ considerably among athletes.

### 4.2. Inter-Individual Differences

**Figure 6:**
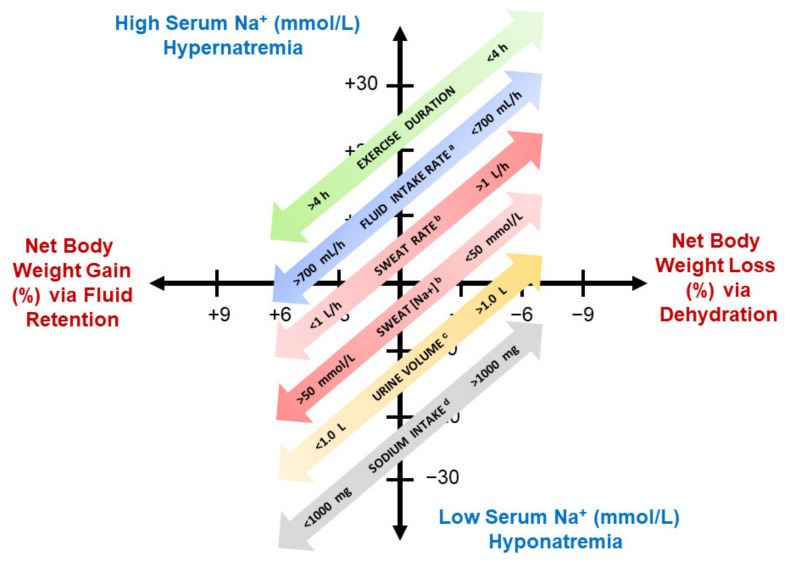
Factors that influence the relationship between body weight gain/loss and serum Na^+^ during endurance exercise. The largest effects on whole body fluid–electrolyte balance are the volume of sweat lost and the volume of fluid consumed. Notes: ^a^, water and low-sodium fluids promote dilution of body fluids; ^b^, physical training and heat acclimatization increase sweat rate and decrease sweat sodium concentration; ^c^, urine production decreases during exercise; ^d^, increased dietary sodium encourages water retention but affects only a minor increase of serum Na^+^**.**

Figure 6 illustrates six factors that influence the change of body mass and serum Na^+^. Interactions of these factors with inherited characteristics and endurance training regimens produce great differences among athletes. In Figure 2, for example, if a male athlete experienced a body mass change of only—0.5% (0.35 kg) during a 13 h Ironman triathlon, his serum Na^+^ could range from 119 (symptomatic EHN) to 157 mmol/L (severe hypernatremia). Conversely, if a male athlete finished with a serum Na^+^ of 140 mmol/L (i.e., in the center of the reference range for healthy adults), his body weight change could range from +5.5% (+3.9 kg) to −12.5% (−8.8 kg). These large serum Na^+^ and body mass ranges represent the effects of numerous perceptual, behavioral, hereditary and fluid-electrolyte variables (Figure 4, Figure 5 and Figure 6), but are influenced mostly by the total volume of sweat produced (e.g., 15.6 L/13 h at a sweat rate of 1.2 L/h) and the total volume of fluid consumed (e.g., 9.1 L/13 h at a drinking rate of 700 mL/h).

Large inter-individual differences also exist among elite runners. Fluid intake rates were determined for 10 men who placed 1st or 2nd (range of finish times, 2:03:59 to 2:10:55) during prestigious city marathons [98]. Each runner’s drinking behavior was recorded on videotape and fluid intake was estimated by multiplying drinking time by 45.2 mL/s (i.e., a value determined by laboratory drinking simulations). Half of these runners consumed fluids at rates that ranged from 30–300 mL/h (73–631 mL total), whereas the rate of fluid intake of the other 5 elite runners ranged from 420–1040 mL/h (886–2205 mL total). Clearly, these data indicate that elite marathon runners (a) ingest fluid at rates that span a wide range during competition (30–1040 mL/h), and (b) some individuals greatly exceed the proposed drinking rate threshold at which symptomatic EHN appears (700 mL/h), described above in Section 3.2.4.

### 4.3. Personal Beliefs and Sources of Rehydration Information

Personal beliefs about drinking may predispose an athlete to EHN, according to the findings of two case reports. The first [66] involved cyclists LC and AM in Table 3 (who also are designated as athletes H and J in Table 4). Their urine specific gravity measurements on Day-1 (LC, 1.006; AM, 1.004; 31 control cyclists, 1.017) and the morning of the road ride (LC, 1.003; AM, 1.005; controls, 1.019) indicated that both had overhydrated before prolonged exercise. The authors suggested that their pre-event hydration behavior, coupled with high rates of fluid intake during the ride (LC, 1500 mL/h; AM, 1400 mL/h), resulted in both cyclists experiencing asymptomatic EHN. The second publication [61] involved a controlled case report of EHN (serum Na^+^, 122 mmol/L) observed during 5 h of intermittent treadmill walking in a hot environment (Tamb, 41 °C). A physically fit, 21 year-old male began exercise with blood values (serum Na^+^, 134 mmol/L; osmolality, 282 mOsm/kg) slightly below the laboratory reference ranges for healthy males, because he had voluntarily overhydrated throughout the previous day. Overhydration prior to exercise is known to lower serum Na^+^ and therefore increase the risk of dilutional hyponatremia if fluids are aggressively consumed during exercise [24]. This man acknowledged that his pre-exercise overhydrated state, and his high rate of fluid intake during exercise (2061 mL/h; 10.31 L total), were purposeful. He believed that drinking a large volume of water would decrease his risk of exertional heat exhaustion and heatstroke.

To understand the factors that guide drinking behavior, Winger and colleagues [99] surveyed 106 female and 97 male runners who had competed in road races (average experience, 13.0 and 8.3 y, respectively) at distances ranging from 5 to 42.2 km. Seven years later, Wilson [100] surveyed 223 female and 199 male marathon runners (average number completed by both groups, 4). Both studies focused on runner perceptions of fluid replacement, their beliefs about rehydration during exercise, and drinking behaviors. Winger et al. [99] observed that the most important influences on the drinking behavior of runners were: trial and error/personal history, recommendations from running groups or clubs, and advice from friends; sport drink companies were the least influential sources of information. Among marathon runners, Wilson [100] reported that the following sources of information were considered to be the most reliable: social media, print magazines, a personal trainer, and a fellow runner; scientific journals, dietitians/nutritionists, and running coaches were rated as the least reliable sources of information.

### 4.4. Unique Characteristics of Competitive Events

Distinctive aspects of different sporting events also can influence an athlete’s rate of fluid intake; three examples follow. First, the possibility of inhaling water when respiratory ventilation rate is high (e.g., 120–155 L/min), and potentially having to stop to clear both lungs [21], may deter runners from drinking. Second, whereas fluid intake during endurance footraces is generally limited to the number of aid stations along a race course or a water bottle in hand, cyclists can consume fluids whenever desired because containers are attached to the bicycle frame or held in jersey pockets. Third, during the water stage of a triathlon, endurance swimmers have no access to fluids except the water they inadvertently consume during competition. These factors explain, in part, the consistent observation that endurance competitors replace ≤ 50% of sweat losses when allowed liquids ad libitum [101,102,103].

## 5. Rehydration Options

Table 5 describes five approaches to rehydration that endurance athletes employ: drink when thirsty, ad libitum drinking, individualized planned drinking, drink nothing, and drink as much as possible. In the following paragraphs, these approaches will be named options 1 through 5. The percentage of athletes who employ each option during endurance exercise is unknown.

### 5.1. Options 1 and 2

The distinction between option 1 (using perceived thirst as the only signal to drink) and option 2 (consuming fluid whenever and in whatever volume desired) is subtle [101]. In fact, some professional organizations and experts have not recognized these as distinct behaviors and some authors use these terms synonymously [6,26,84,98,99,101]. However, a 2014 field study determined that cyclists could identify whether they typically used option 1 or 2 and self-selected into one of two study groups (*n* = 12 in each). Despite the fact that cyclists understood options 1 and 2 as distinct rehydration behaviors, no between-group differences were observed in the following measurements: total fluid intake (L), body mass change (%), time to ride 164 km (h), urine specific gravity, and rating of thirst at the finish line [110]. Thus, the greatest value of distinguishing options 1 and 2 may exist in communications among athletes.

During prolonged endurance exercise, the effectiveness of thirst and drinking in maintaining whole-body fluid–electrolyte balance is challenged [32] by large water and sodium losses in sweat, perturbations of intracellular volume or concentration, and plasma volume depletion [6]. As one example of several in the literature, Greenleaf et al. [91] reported that test subjects who drank ad libitum during exercise-heat stress, were not thirsty and felt fully recovered despite a body water deficit of 4–5 L. This phenomenon was named involuntary dehydration [6,12] because thirst was not sufficient to maintain body water and fluid intake did not match the level of dehydration. Voluntary dehydration is observed in most endurance athletes [17,102].

A field study involving 26 ultraendurance cyclists explored thirst during exercise [114]. Ratings of thirst were statistically compared to several other variables during 7 h of exercise (mean ground speed, 25.4 km/h; Tamb, 35.5 °C). The total fluid intake of these male cyclists varied greatly, ranging from 2.1 to 10.5 L during the 164 km ride. Post-event ratings of thirst were not significantly correlated with any measured variable, including total fluid intake (i.e., a measure of drinking behavior), body mass index, height, ground speed, body water balance (ingested fluid volume—volume of fluid lost), and change of body mass. In other words, the intensity of thirst did not represent the degree of dehydration or the volume of fluid consumed. This observation is consistent with the known effects of oropharyngeal sensations on drinking behavior. These sensory signals (e.g., mouth dryness, swallowing fluid) rapidly reduce and limit fluid intake by modulating satiety and opposing overdrinking [115]. Thus, whenever fluid is consumed, even if the volume is small, oropharyngeal signals diminish the sensation of thirst [93,115] and theoretically reduce the risk of EHN by reducing fluid intake.

### 5.2. Option 3

Individualized planned drinking appeals to athletes who prefer to design their exercise rehydration systematically. This approach to rehydration is recommended by the National Athletic Trainer’s Association [26] and the American College of Sports Medicine [24]. The accuracy of this method relies on an objective measurement of sweat rate during outdoor exercise. After sweat rate is determined, the athlete can design a customized fluid replacement plan for use during endurance exercise, realizing that the predetermined rate of fluid intake always should be less than sweat rate, to avoid overhydration and body weight gain.

#### 5.2.1. Determining Sweat Rate

The method to determine sweat rate involves voiding the bowel and bladder, weighing body mass before exercise on a digital floor scale with a precision of 0.1 kg (i.e., 0.2 lb), simulating a future competitive event (i.e., considering environmental conditions, exercise intensity), and measuring body weight after exercise [116,117]. Sweat rate equals the body mass change per hour. If fluid is consumed or if urine is excreted between the pre- and post-exercise body weight measurements, the final sweat rate should be corrected as follows: sweat rate (L/h) = body weight difference (1 kg = 1 L) + water intake (L)—urine volume (L). All factors are measured over a 1-h or half-hour period; the latter is corrected to 1 h mathematically.

#### 5.2.2. Determining a Morning Baseline Body Mass

As body mass measurements may be impractical or impossible at the event site, it is valuable for each athlete to determine his/her baseline body mass approximately 1 week before an event. This is done by measuring body weight upon waking, on 3–5 consecutive days, using an accurate digital floor scale (±0.1 kg or lb). The median (middle) or average body mass serves as a useful baseline [116]. The important comparison is made between this baseline value and the body mass measured on the morning after an endurance event [118,119]. If the post-event body mass is notably less or greater than baseline, fluid intake should be adjusted accordingly (i.e., avoiding overconsumption and underconsumption) for 1–2 days; thirst and urine color changes will assist this adjustment of fluid intake [5,118,119]. Three other details are important. First, wear little or no clothing each time that body mass is recorded. Second, if dietary carbohydrate “loading” is used during the days prior to competition, body mass may increase (0.5–1.5 kg) because water is stored with glycogen in skeletal muscles. This extra water temporarily inflates body mass measurements, and these values should not be used to determine one’s baseline. Third, this technique may not be valid after an event lasting 7–24 h because loss of muscle mass and/or fat mass may confound interpretation of body water status for several days after an ultraendurance event [16].

#### 5.2.3. Interpreting Body Mass Changes

During exercise lasting 0.5–4.0 h, body mass change (ΔM_b_) is the most commonly used representation of body water change [120,121] because most of body weight loss is water loss, muscle and adipose tissue losses are negligible, and ΔM_b_ has a measurement resolution of ±0.1 L (e.g., 100 mL out of a 42–47 L total body water volume) when using a floor scale that reads to ±100 g. Within the time frame of 0.5–4.0 h, ΔM_b_ essentially equals water loss (i.e., when corrected for the mass of fluid and food intake, urine output, and sweat loss), because no other body constituent is lost at a similar rate [120,122]. Option 3 in Table 5 is the only approach to rehydration that incorporates a known sweat rate value (calculated using ΔM_b_ measurements) and a planned rate of fluid intake (personally monitored during competition).

When endurance exercise lasts longer than 4 h, the contributions of other factors confound the interpretation of ΔM_b_ [13]. These include mass loss due to carbohydrate or fat oxidation, cellular water generated during metabolic reactions, and skeletal muscle and/or adipose tissue catabolism [15,16,71]. However, because athletes seldom know the technical methods to calculate these internal gains and losses of water, measurements of ΔM_b_ remain the only realistic surrogate measure of dehydration for athletes and field-based practitioners [15,77,119,122].

### 5.3. Options 4 and 5

The practices of drinking as much as possible (i.e., increasing the risk of EHN) and drinking nothing (i.e., increasing the likelihood of a performance decrement) are discouraged. No professional sports medicine or sports nutrition organization recommends these extreme options during endurance exercise.

## 6. Rehydration Recommendations for Endurance Athletes

Attempting to control all of the factors that influence thirst, drinking behavior, exercise performance, and health (described in the paragraphs above) is a formidable and unreasonable task. Nevertheless, the aim of rehydration should be to consume a volume of fluid that not only avoids dehydration greater than 2–4% of body mass, but also avoids overhydration. Although no single recommendation will suffice for all individuals (e.g., across a range of ambient temperatures, and with varied sweat rates, body masses, and exercise durations/intensities) [123], the following 9 recommendations are appropriate for most endurance and ultraendurance activities.

1. Measure body weight before and after exercise (Section 5.2.1). Change of body mass during exercise is a reasonable, albeit not perfect, surrogate measure of water gain or loss [15,77,119,122]. If body weight cannot be assessed on the day of endurance exercise, measure body weight on the morning after and compare this weight to a pre-determined baseline morning body weight [119]. Detailed methods are described above in Section 5.2.2 and Section 5.2.3.
2. Do not gain weight during endurance exercise. Weight gain typically indicates fluid retention and increased risk of EHN [32,63].
3. Consume fluid at a rate less than 700 mL/h to reduce the risk of EHN. The proposed rationale for this recommendation is described in Section 3.2.4, Table 4 (column 8, symbols A–E), and Figure 2 (gray and yellow highlighted symbols). This recommendation is consistent with the 2001 guidelines of the International Marathon Medical Directors Association [84], and the 2007 fluid replacement position stand of the American College of Sports Medicine [24]. Both organizations recommend a 400 to 800 mL/h rate of fluid intake during endurance exercise.
4. Be alert for physiologic and perceptual cues that discourage drinking. When stomach fullness, bloating, or vomiting are experienced, decrease fluid intake [84].
5. Guide drinking behavior with this in mind: modest levels of dehydration up to 2–3% of body weight are tolerated well, with little risk of morbidity, symptoms, or a decline in exercise performance (Figure 1; Figure 3) [77].
6. According to the International Marathon Medical Directors Association [84], a weight loss that exceeds 4% of body mass justifies a medical consultation. The number of athletes affected by at least a 4% loss of body mass during prolonged endurance exercise is considerable, as shown in Figure 2.
7. After endurance exercise, white salt deposits on a shirt, jersey, or shorts indicate both a high sweat rate and a high sweat sodium concentration [124]. Consecutive days of profuse sweating (e.g., during lengthy training sessions) or a day-long utraendurance event in a hot environment may lead to whole-body salt deficiency [125,126] due to large sweat sodium losses, inadequate sodium intake, or both [46]. If salt depletion is suspected (e.g., increased salt appetite or salt craving), it is prudent to consider adding specific dietary food items to ensure that daily sodium intake replaces exercise-induced sodium loss. Refer to [97] to identify the amount of sodium in common food items. Sodium supplementation during meals should be guided by dietary recommendations for daily sodium intake [127], and by considering the potential negative health effects of chronic high dietary salt intake [128].
8. Sodium consumption in solid food or capsules has a minor influence on serum Na^+^ and whole-body sodium balance during endurance exercise (Section 3.2.5) [88,89]. Athletes should be aware that sodium intake, while not discouraged, may provide little or no defense against EHN during prolonged exercise and the effects are unpredictable (see Table 3). This recommendation is supported by observations of ultramarathon runners [80]. Multiple regression analysis indicated that the amount of sodium consumed during a 161 km race accounted for only 6–8% of the variance in post-race serum Na^+^. This recommendation also is supported by the Wilderness Medical Society Clinical Practice Guidelines [123], which advise that sodium and/or salty snacks be consumed along with an appropriate fluid volume. Salt intake should not be combined with overdrinking, which increases the risk of EHN despite sodium consumption; see recommendations 2–4 above.
9. Experiment with rehydration options (Table 5) during training sessions, before using them in competition or in hot environments.

## Figures and Tables

**Table 1:** The relative effects of thirst, drinking, and physiological responses on fluid-electrolyte balance during ordinary daily activities and endurance exercise.

Activity	Thirst & Drinking Behavior	Sweat Gland Secretion of Hypotonic Fluid	Kidney Regulation of Water & Electrolytes	Neuroendocrine Homeostatic Responses ^a^	Effects on Water & Electrolyte Balance
Sedentary daily activities (16 h)	Basal ^b^	Negligible	Basal ^b^	Basal ^b^	CNS responses are sufficient to maintain water and electrolyte homeostasis
Brief exercise (5–30 min) at moderate-to-high intensity	Minor	Minor-to-moderate	Minor	Minor, brief	Water and electrolyte losses are minor
Endurance exercise (0.5–5 h) at low-to-high intensity	Minor-to-large	Moderate-to-large	Minor-to-moderate	Minor-to-large, prolonged	Moderate-to-large turnover ^c^ due to sweating and drinking
Ultraendurance exercise (5–24 h) at low-to-moderate intensity	Moderate-to-large	Large	Moderate-to-large	Large, prolonged	Water and electrolyte losses in sweat and urine exceed 24 h dietary intake

CNS, central nervous system (i.e., brain and spinal cord); TBW, total body water. ^a^, CNS effects involving nerves and hormones that regulate whole-body water volume and concentration, blood volume/pressure/osmolality, and thirst (see [9] for a review of this topic). ^b^, a standard low level maintenance of whole-body fluid-electrolyte balance with small turnover (intake versus loss) and minor perturbations. ^c^, turnover refers to the sum of gains and losses of water and electrolytes.

**Table 2:** Characteristics of three groups of cyclists who completed a 164 km summer road cycling event in 4.8–9.6 h (modified from [15]). No drinking instructions or experimental interventions were involved.

Variables	Average Exercise Duration (h) ^a^		
	9.6	6.3	4.8
Pre-event body mass ^b^ (kg)	81.90	82.05	82.55
Number of male cyclists	11	11	10
Ground speed (km/h)	17.2 ^d^	26.6 ^d^	34.0 ^d^
Rating of perceived exertion at finish ^c^	16	16	16
ad libitum total fluid intake ^e^ (g)	+6100	+4500	+3900 ^f^
Rate of fluid intake (g/h)	+635	+715	+810
Sweat secreted ^g^ (g)	−7700	−7150	−7000
Sweat rate (g/h)	−800 ^d^	−1135	−1460
Urine excreted ^g^ (g)	−1300	−550	−450 ^d^
Solid food mass consumed ^e^ (g)	+423	+355	+350
Body mass change ^b^ (g)	−1800	−2300	−2750
Body mass change (%)	−2.0	−2.9	−3.4

Note: values are means or medians; negative values represent reduced mass or loss of fluid from the body; air temperature ranged from 24.4 °C (08:00 h) to 41.1 °C (15:00 h); for the purposes of this table, 1 g = 1 mL and 1 kg = 1 L. ^a^, cyclist groups 9.6 and 6.3 voluntarily stopped at 3 roadside aid stations for research measurements, elimination, drinking, and eating. Group 4.8 rode as part of a 5-h pace team and did not stop during the entire event. ^b^, measured with a calibrated floor scale (±100 g). ^c^, using a printed 6 (very, very light) to 20 (very, very hard) point perceptual rating scale [19]. ^d^, significantly different from all other groups (*p* = 0.01 to 0.0001). ^e^, based on cyclist diet records and confirmed by interviews. ^f^, significantly different from group 9.6 (*p* = 0.04). ^g^, detailed methods are described in the original publication [15].

**Table 3:** Athlete physiological and perceptual responses during a summer road cycling event (7.1–10.9 h duration). Data are rank-ordered on the basis of serum Na^+^ change (column 5). Modified from [66] with unpublished data added.

Cyclists	Total Fluid Intake (L) ^a,b^	Total Fluid Intake (ml/kg) ^a,b^	Sodium Intake (mg) ^a,b^	Change of Serum Na^+^ (mmol/L) ^a^	Pre-Event Body Mass (kg)	Body Mass Change (%) ^a^	Urine Specific Gravity at Finish Line	Rating of Thirst at Finish Line ^c^	Environmental Symptoms Questionnaire ^d^ Total Score at Finish Line
A	3.7	42	356	+6	88.6	−4.6	1.021	4	13
B	5.3	75	194	+4	71.0	+1.4	1.024	8	10
C	3.0	48	328	+3	61.8	−4.2	1.030	6	11
D	4.7	62	149	+1	75.2	−1.2	1.026	8	27
E	10.9	139	1166	+1	78.5	−1.5	1.020	7	25
F	4.6	54	124	−1	85.5	+0.1	1.021	6	21
G	4.1	50	261	−2	82.0	−1.8	1.030	5	13
H	3.4	41	263	−2	82.9	−0.1	1.023	4	11
I	9.5	103	823	−2	91.8	−4.6	1.034	6	25
J	9.6	124	1259	−3	77.2	−1.9	1.016	4	17
K	10.5	101	1182	−3	104.7	+1.0	1.026	5	21
L	9.2	109	1601	−6	84.7	+1.1	1.003	5	12
LC ^e^	13.7	191	1179	−11	72.0	+4.3	1.003	2	4
AM ^e^	14.7	189	3292	−11	77.5	+0.1	1.010	2	11

^a^, during the 164-km ride; ^b^, consumed in water, beverages, sport drinks, solid foods, bars, gels, tablets, capsules; ^c^, a visual rating scale presented thirst levels of increasing intensity, ranging from 1(not thirsty) to 9 (very, very thirsty); ^d^, see reference [65]; ^e^, cyclists LC and AM experienced exertional hyponatremia, both with a serum Na^+^ of 130 mmol/L.

**Table 4:** Factors that influence exertional hyponatremia. Letters in column 1 refer to the symbols embedded in Figure 2.

Symbols in Figure 2	Men	Women	Scenario (Ambient Temperature, °C)	Final Serum Na^+^ (mmol/L)	Body Mass Change (%)	Exercise Duration (h)	Rate of Fluid Intake (ml/h)	Mean Initial Body Mass (kg)	Source
Background data points	^a^	^a^	11 endurance events ^a^	See Figure 3	See Figure 3	^b^	^b^	^b^	[20]
A	42		164 km cycling (34.4)	141	−0.8	9.1	649	85.9	[55]
B	31		164 km cycling (24.4–39.5)	141	−1.4	9.0	700	85.4	[66]
C		6	164 km cycling (34.4)	140	−0.1	9.0	520	67.3	[55]
D	50		100 km run (15.6–21.7)	138	−2.6	12.2	600	74.9	[71]
E	7		Treadmill walk (41.0) ^c^	136	−0.1	4.0 ^c^	640	77.9	[61]
F	5		44 km trail run (15–34)	131	−2.2	9.3	290 ^d^	81.9	[72]
G		1	Ironman triathlon (21.0) ^e,f^	131	+0.9	13.3	733	57.5	[73]
H	1		164 km cycling (24.4–39.5) ^g^	130	+4.3	8.9	1,500	72.0	[66]
I		1	Ironman triathlon (21.0) ^e,f^	130	+2.5	12.0	764	59.0	[73]
J	1		164 km cycling (24.4–39.5) ^g^	130	+0.1	10.6	1,400	77.5	[66]
K	2	5	Ironman triathlon ^e^	128	−0.5	12.3	^b^	62.5	[74]
L	1		Treadmill walk (41.0) ^c^	122	+4.0	4.0 ^c^	2,061 ^h^	82.2	[61]
M	1		Ironman triathlon ^e^	116	+5.0	14.0	1,642	^b^	[75]

Note: values are means or medians (columns 5–9) when the number of subjects is ≥ 2. ^a^, 3 Ironman triathlons, 6 marathon footraces (42.2 km), a 109 km cycling tour, and a 160 km footrace (2,135 athletes); ^b^, not reported; ^c^, 5.6 km/h, 5% grade, 30 min walking and 30 min seated rest per hour; ^d^, runners were allowed to drink and eat only fluids and food provided by the race organizing committee, at 11 intermediate checkpoint stations, positioned every 3–5 km; they did not drink when thirsty or ad libitum; ^e^, triathlon stages were 3.8 km swim, 180 km cycle, 42.2 km run; ^f^, this athlete stopped during the cycling stage due to hyponatremic illness; ^g^, identical to cyclists LC and AM in Table 4 (column 1); ^h^, this individual purposefully overhydrated.

**Table 5:** Five options for rehydration during endurance exercise.

Description	Objective/Rationale	Relevant Publications
1. Drink when thirsty. Fluid intake occurs only when thirst is sensed.	Primary focus: to prevent exertional hyponatremia. Secondary goal: to prevent a level of dehydration that impairs exercise performance. Proponents of this method assert that increased extracellular concentration triggers thirst to naturally protect athletes from the negative consequences of both fluid excess and severe dehydration. However, no randomized, controlled study confirms that drinking when thirsty successfully prevents exertional hyponatremia. Rationale: drinking when thirsty preserves serum Na^+^ and osmolality within the normal laboratory reference range.	[55,84,104,105,106,107,108,109,110]
2. Ad libitum drinking. Consuming fluid whenever and in whatever volume desired, without specific focus on thirst.	Primary focus: to prevent exertional hyponatremia. Secondary goal: to prevent a level of dehydration that impairs exercise performance. Ad libitum drinking often is viewed as being identical to drinking when thirsty (above), however it is subtly different. See text for details.	[6,55,101,110,111]
3. Individualized planned drinking. This involves drinking a predetermined fluid volume that is determined by measuring sweat rate.	Primary focus: to prevent excessive dehydration that impairs exercise performance and to prevent exertional hyponatremia. Secondary goals: to decrease the risk of heat illness (heat exhaustion, heat stroke), and reduce cardiovascular/thermoregulatory strain associated with dehydration. Rationale: because there is considerable inter-individual variability of sweat rate and sweat electrolyte concentration, a customized fluid replacement plan meets each athlete’s individual rehydration needs.	[24,26,32,66,108,112,113]
4. Purposefully drink nothing during exercise.	No professional sport medicine or sport nutrition organization recommends this extreme option for prolonged endurance exercise.	
5. Purposefully drink as much as possible, in excess of thirst.	No professional sport medicine or sport nutrition organization recommends this extreme option for prolonged endurance exercise. Nevertheless, a 2011 survey of runners (5 to 42.2 km finishers) determined that 8.9% plan to drink as much as possible during racing and training.	[99]

## Data Availability

No new data were created or analyzed in this study. Data sharing is not applicable to this article.
